# Dynamic Patterns and Predominance of Respiratory Pathogens Post-COVID-19: Insights from a Two-Year Analysis

**DOI:** 10.1007/s44197-024-00213-9

**Published:** 2024-04-08

**Authors:** Salma AlBahrani, Samira Jamaan AlZahrani, Thekra N. Al-Maqati, Atheer Almehbash, Anfal Alshammari, Refan Bujlai, Sarah Ba Taweel, Fares Almasabi, Abdullah AlAmari, Jaffar A. Al-Tawfiq

**Affiliations:** 1 Infectious Disease Unit, Specialty Internal Medicine, King Fahd Military Medical Complex, Dhahran, Saudi Arabia; 2 College of medicine-Imam Abdulrahaman Bin Faisal University, Dammam, Saudi Arabia; 3 Molecular laboratory department, King Fahd Military Medical Complex, Dhahran, Saudi Arabia; 4 Department of Clinical laboratory Science, Prince Sultan Military College of health science, Dammam, Saudi Arabia; 5 Radiology Department, Armed Forces Hospital Najran, Najran, Saudi Arabia; 6 Department of pharmacy, King Fahd Military Medical Complex, Dhahran, Saudi Arabia; 7 Infectious Disease Unit, Specialty Internal Medicine, Johns Hopkins Aramco Healthcare, Dhahran, 31311 Saudi Arabia; 8 Infectious Disease Division, Department of Medicine, Indiana University School of Medicine, Indianapolis, IN USA; 9 Infectious Disease Division, Department of Medicine, Johns Hopkins University School of Medicine, Baltimore, MD USA

**Keywords:** COVID-19, Respiratory pathogens, RSV, Influenza, SARS-CoV-2

## Abstract

**Introduction:**

Respiratory tract infections (RTIs) stand out as the most frequent causes leading to visits to the emergency department and hospitalizations. This study aims to assess the types and prevalence of respiratory infections across two years following the end of the COVID-19 pandemic.

**Methods:**

Patients presenting with an influenza-like illness (ILI) were tested using multiplex RT-PCR (QIAstat-Dx, Qiagen). The multiplexed RT- PCR test detects 21 respiratory viruses and bacteria.

**Results:**

During the study period, PCR test was done on a total of 1,790 samples were tested, and 712 (40%) were positive for a total of 796 pathogens. The mean age (± SD) of the participants was 20.1 ± 28.4 years in 2022 and 21.9 ± 27.6 years in 2023. Among the detected pathogens, the most prevalent were Rhinovirus/Enterovirus 222 (12.4%), followed by RSV A&B (103 cases, 5.7%), and H1N1 Influenza (77 cases, 4.3%). Additionally,  Influenza A/B constituted 172 (9.6%) while parainfluenza constituted (58, 3.2%). SARS-CoV-2 was identified in 3.97% of the samples. Over the two-year period, the monthly pattern of the identified pathogens exhibited fluctuations in the prevalence. Furthermore, variations were observed in the detected pathogens across different age groups.

**Conclusion:**

In addition to adding significant knowledge to the field of respiratory viral infections, this study emphasizes the necessity of ongoing research and surveillance for the detection and characterization of respiratory viruses, particularly those with the potential for emergence. Such studies would also require setting up a strategy for genotyping and/or sequencing of viruses.

## Introduction

Respiratory tract infections (RTIs) are among the most prevalent illnesses that cause a high number of visits and hospital stays, and they have a significant morbidity and mortality rate, particularly in younger children and the elderly [1]. Rhinovirus type A and C, as well as respiratory syncytial virus (RSV), have been linked to chronic lung illnesses such asthma [2–4]. The prevalence of various RTIs tends to follow distinct seasonal trends [5, 6]. Seasons with the highest prevalence of viral infections are typically the fall and winter because temperature and humidity, for example, have an effect on the stability and rates of transmission of respiratory viruses as well as the host’s immune responses to RTIs [7, 8]. However, a key element in the propagation of viruses is the patterns of human behavior, which affects the frequency of interaction between susceptible hosts and infected persons. The COVID-19 pandemic significantly reduced contact rates due to social interactions, mask wearing, and hand hygiene [9, 10]. During the pandemic, these non-pharmaceutical interventions (NPIs) greatly reduced the transmission of viruses, such as SARS-CoV-2, RSV, and influenza A/B [9, 10].

Following the easing of pandemic-related limitations and decreased adherence to NPIs, a growing and persistent wave of non-COVID-19 RTIs in children has been seen since June 2021 [11–14]. Variations in the respiratory virus population in communities have been associated with viral interference and virus-virus interactions [15]. Variation in adherence to NPIs at different levels such as the community, regional, and national levels, varied especially after lifting the COVID-19 restrictions. These differences may have affected how respiratory pathogens disseminate within any given community. Although several European countries reported an increase in RTIs in 2022, it is unclear if coinfection frequency and clinical significance changed from the pre-pandemic period [2]. There are limited data from Saudi Arabia about the pattern of RTIs and causative agents in the post-COVID-19 era [16]. In Saudi Arabia, the COVID-19 pandemic had caused 837,569 cases including 9,646 (1%) deaths. The country had four main waves: the first wave peaked in June 2020, the second wave peaked in June 2021, the third peaked in January 2022, and the fourth peaked in June 2022. There were differences in these waves in relation to the number of cases as well as the death rates [17–19]. The third wave coincided with the Omicron variant and had caused a large number of cases but a lower mortality [19, 20]. One study from Saudi Arabia showed a significantly lower number of RSV cases admitted in 2022 compared to 2019, and this was attributed to the application of non-pharmacologic interventions to reduce COVID-19 cases [16]. In the Middle East and North Africa (MENA) region, a systematic review showed higher number of RSV during the winter months from November to February [21]. The full picture of the pattern of the circulating pathogens causing RTIs in Saudi Arabia is sparse [22]. Most of the studies had specifically addressed the pattern among Pilgrims and the Middle East respiratory Syndrome Coronavirus (MERS-CoV). Other studies had addressed specific pathogens such as RSV and human respiratory syncytial virus. Thus, to enhance our understanding of the epidemiology of RTIs post-COVID-19, this study intends to look into the patterns of pathogens of RTIs from 2022 to 2023 in a hospital in Saudi Arbia.

## Materials and methods

We conducted a retrospective cohort study in a hospital in Saudi Arabia, between January 2022 and November 2023. Patients presenting with an influenza-like illness (ILI) were tested using multiplex RT-PCR (QIAstat-Dx, Qiagen). The test was performed on swab samples from the upper respiratory tract (nasopharyngeal). The QIAstat-Dx Analyzer is a fully automatic diagnostic device and performs a multiplexed RT- PCR test for the detection of 21 respiratory viruses and bacteria. These pathogens are *Mycoplasma pneumoniae, Chlamydophila pneumoniae, Bordetella pertussis*, Influenza A, Influenza A subtype H1N1/2009, Influenza A subtype H1, Influenza A subtype H3, Influenza B, Parainfluenza virus 1, Parainfluenza virus 2, Parainfluenza virus 3, Parainfluenza virus 4, SARS-CoV-2, Coronavirus 229 E, Coronavirus HKU1, Coronavirus NL63, Coronavirus OC43, Adenovirus, RSV A/B, Human Metapneumovirus A/B, and Rhinovirus/Enterovirus, as described previously [23, 24]. The study included any patient who presented with ILI and was tested for the presence of respiratory pathogens. Confirmed cases were those who tested positive using real-time PCR. The study received approval from the institutional board (IRB).

Statistical Analysis: We classified participants with a positive PCR test into different age groups: infants and toddlers (< 2 years), early childhood (2–5 years), middle childhood (6–11 years), early adolescence (12–18 years), young adulthood (19–44 years), middle adulthood (45–64 years), and older adulthood (65 years and older) [25]. We examined the rate of occurrence of different pathogens in each group to allow for a comprehensive understanding of the impact of respiratory pathogens across the lifespan. We summarized the characteristics of continuous and categorical data as numbers and percentages. Statistical analysis was performed using the JASP  [Computer software] (Version 0.18.1). A P value of ≤ 0.05 was considered as statistically significance.

## Results

During the study period, a total of 1790 patients with ILI were tested and 712 (40%) were positive, yielding a total of 796 pathogens. The overall mean age (± SD) was 20.1 ± 28.4 years (Table 1) with a male to female ratio of 1.1:1. The analysis of the samples collected over a two-year period revealed a diverse range of respiratory pathogens.  Among these, the most prevalent pathogen was Rhinovirus/Enterovirus detected in 222 samples  (12.4%).  This was followed by RSV A&B found in  103 samples (5.7%), and Influenza A H1N1 detected in 77 samples (4.3%) (Table 2). Influenza A/B constituted 172 (9.6%) and parainfluenza accounted for  58 samples (3.2%). Notably, SARS-CoV-2 was identified as the fourth leading pathogen present in  71 (4%) during this period. Several other respiratory agents were detected at relatively low frequencies, including *Mycoplasma pneumoniae*, and *Legionella pneumophila*.

**Table 1:** Association of Pathogen Epidemiology with Age and Sex

	Number			Age			
	Total	Female	Male	Mean	Std. Deviation	Minimum	Maximum
229E	4	1	3	60.5	30.8	30	103
Adenovirus	15	5	10	10.9	17.6	0.66	71
BOCA	17	9	8	2.5	1.4	1	6
SARS-CoV-2	66	36	30	45.3	27.8	0.66	94
FLU A	55	27	28	33.6	27.3	0.08	97
FLU B	28	12	16	19.8	20.2	1	63
H1N1	74	39	35	34.4	27.8	0.08	90
HKU1	2	1	1	28.0	38.2	1	55
HMPV	46	25	21	24.4	28.3	0.83	83
Mycoplasma	2	2	0	21.5	29.0	1	42
NL63	8	2	6	23.5	22.3	1	60
OC43	17	8	9	38.3	31.6	0.4	91
Parainfluenza	44	19	25	26.8	31.8	0.08	96
Rhinovirus	177	77	100	24.8	28.2	0.08	103
RSV	81	38	43	13.6	25.3	0.08	84
Multiple pathogens	76	38	38	10.0	19.8	0.83	88

**Table 2:** Respiratory Test Results for Patients with Influenza-Like Illness

Respiratory Pathogen Detected	Total (%)
Rhinovirus/Enterovirus	222 (12.4)
Respiratory syncytial virus A&B	103 (5.8)
Influenza A H1N1	77 (4.3)
SARS-CoV-2	71 (4.0)
Influenza A H3	58 (3.2)
Human metapneumovirus A&B	55 (3.1)
Adenovirus	47 (2.6)
Parainfluenza virus 3	41 (2.3)
Influenza B	30 (1.7)
Bocavirus	26 (1.5)
Coronavirus OC43	22 (1.2)
Parainfluenza virus 4	9 (0.5)
Coronavirus 229E	7 (0.4)
Coronavirus NL63	7 (0.4)
Parainfluenza virus 1	5 (0.3)
Influenza A (no subtype)	5 (0.3)
Coronavirus HKU1	3 (0.2)
Parainfluenza virus 2	3 (0.2)
Influenza A H1	2 (0.1)
*Mycoplasma pneumoniae*	2 (0.1)
*Legionella pneumophilia*	1 (0.1)
*Bortedettla pertusis*	0 (0.0)
None detected	994 (55.5)

**Fig. 1:**
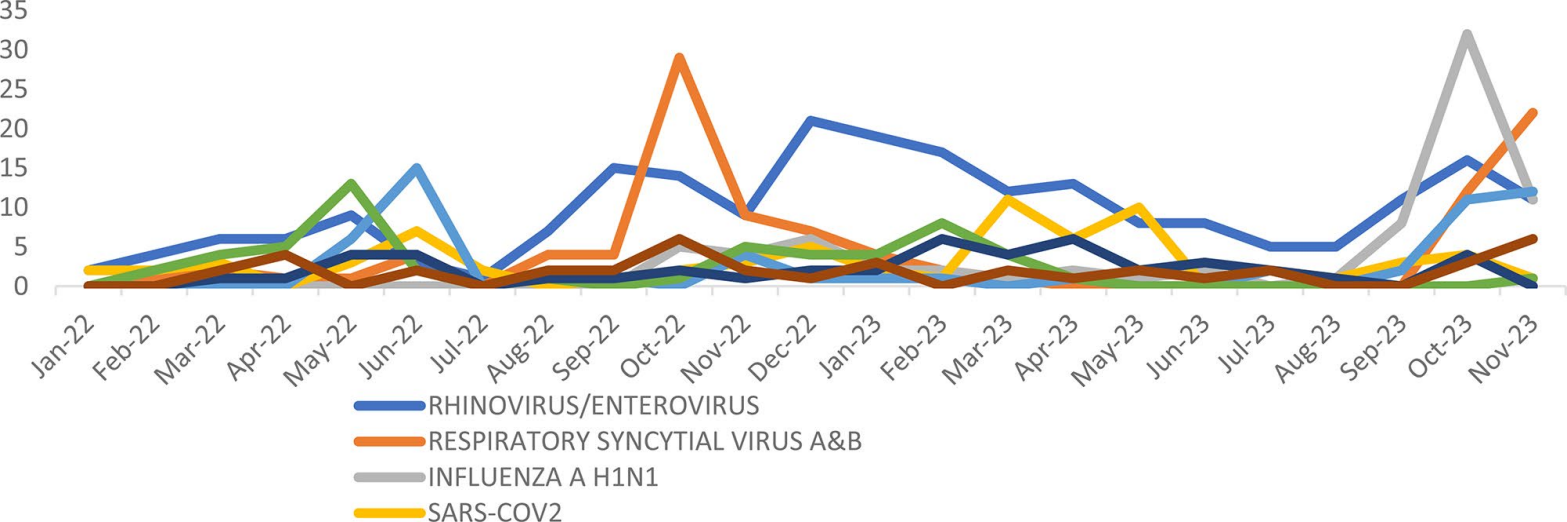
Monthly Trends in the Prevalence of Commonly Detected Respiratory Viruses over the Study Period

The monthly pattern of the top 8 identified pathogens exhibited variations in the number of cases throughout the study period (Fig. 1). For instance, the detection levels of Rhinovirus/Enterovirus varied, with higher counts in September and December 2022. RSV A&B reached its peak in October 2022 and November 2023. SARS-CoV-2 cases were notably higher in June and December 2022 as well as in March and May 2023. Influenza A H3 exhibited an increase in June 2022 and November 2023, while, Influenza H1N1 had its peak in June 2022 and November 2023.

**Fig. 2:**
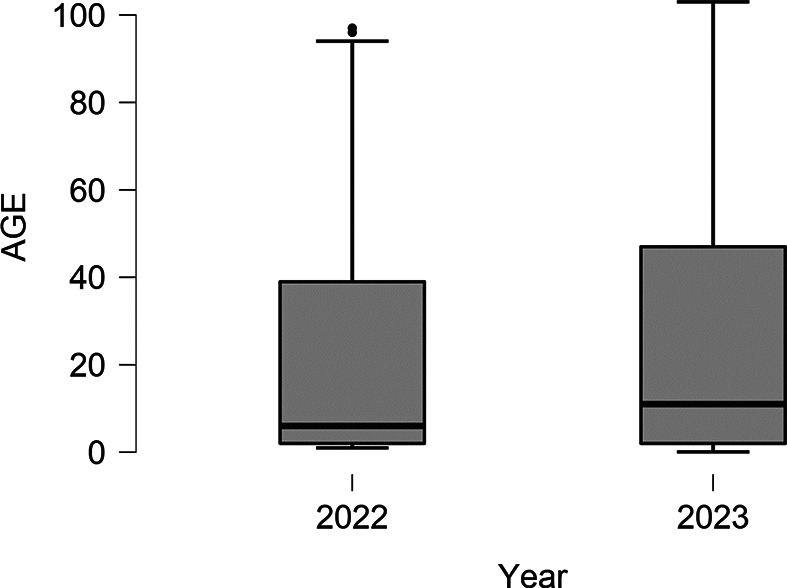
A Comparison of Patient Age Distribution between 2021 and 2022: Boxplots Analysis

 This study aimed to assess the prevalence of respiratory viruses across different age groups (Table 3). The top three pathogens showed variation across the different age ranges. For infants and toddlers (0–2 years), the most prevalent pathogens were RSV with 40 cases (27.6%), followed by influenza B with 12 cases (7.5%), and Rhinovirus with 34 cases (23.4%). In early childhood (3–5 years), RSV was still prominent with 20 cases (12.5%), followed by influenza A with 5 cases (3.1%) and Rhinovirus with 37 cases (23.1%). Among middle childhood (6–12 years), Rhinovirus was the most common with 19 cases (27.9%), followed by RSV with 6 cases (8.8%), and Influenza H1N1 with 10 cases (14.7%). Regarding early adolescence (13-17 years), Rhinovirus was the predominant pathogen, accounting for 15 cases (38.5%). Influenza A and H1N1 followed closely with 5 cases each, representing 12.8% of the cases for both pathogens.  In young adulthood (18–25 years), Rhinovirus remained prevalent with 26 cases (23.4%), followed by SARS-CoV-2 with 19 cases (16.8%), and Influenza H1N1 with 20 cases (18.0%). In middle adulthood (26–64 years), Rhinovirus still ranked first with 16 cases (21.1%), while SARS-CoV-2 had 7 cases (4.8%), and H1N1 had 9 cases (11.8%). Lastly, among older adults (65 + years), Rhinovirus was the most common with 30 cases (26.5%), followed by RSV with 9 cases (8.0%), and Influenza H1N1 with 16 cases (14.2%). The mean age group across the different pathogens was significantly different (*P* < 0.001, in ANOVA analysis) (Table 3). When comparing the mean age of patients in 2022 and 2023 , there was no statistical difference.   The mean age inn 2022 was 21.9 ± 27.6, while in 2023, it was 25.7 ± 28.5 (Fig. 2).

**Table 3:** Prevalence of Predominant Pathogens Across Different Age Groups

Pathogen	Infant and Toddler	Early Childhood	Middle Childhood	Early Adolescence	Young Adulthood	Middle Adulthood	Older Adult
229E	0 (0.0)	0 (0)	2 (2.6)	0 (0.0)	1 (0.9)	0 (0.0)	0 (0.0)
ADENO	5 (3.1)	1 (2.6)	0 (0.0)	1 (2.6)	1 (0.9)	3 (2.1)	4 (5.9)
BOCA	12 (7.5)	0 (0)	0 (0.0)	0 (0.0)	0 (0.0)	4 (2.8)	1 (1.5)
SARS-CoV-2	1 (0.6)	5 (12.8)	16 (21.1)	5 (12.8)	19 (16.8)	7 (4.8)	1 (1.5)
FLU A	7 (4.4)	5 (12.8)	4 (5.3)	5 (12.8)	19 (17.1)	4 (2.8)	12 (10.6)
FLU B	8 (5.0)	1 (2.6)	6 (7.9)	1 (2.6)	5 (4.5)	2 (1.4)	0 (0.0)
H1N1	6 (4.1)	8 (5.0)	10 (14.7)	5 (12.8)	20 (18.0)	9 (11.8)	16 (14.2)
HKU1	0 (0.0)	0 (0)	1 (1.3)	0 (0.0)	0 (0.0)	1 (0.7)	0 (0.0)
HMPV	6 (4.1)	15 (9.4)	5 (7.4)	1 (2.6)	6 (5.4)	6 (7.9)	7 (6.2)
MYCOPLASM	1 (0.7)	0 (0)	0 (0.0)	0 (0.0)	1 (0.9)	0 (0.0)	0 (0.0)
NL63	2 (1.4)	1 (0.6)	0 (0.0)	1 (2.6)	2 (1.8)	2 (2.6)	0 (0.0)
OC43	2 (1.4)	3 (1.9)	0 (0.0)	0 (0.0)	4 (3.6)	3 (3.9)	5 (4.4)
Parainfluenza	8 (5.5)	11 (6.9)	6 (8.8)	2 (5.1)	4 (3.6)	5 (6.6)	8 (7.1)
RHINO	34 (23.4)	37 (23.1)	19 (27.9)	15 (38.5)	26 (23.4)	16 (21.1)	30 (26.5)
RSV	40 (27.6)	20 (12.5)	6 (8.8)	0 (0.0)	2 (1.8)	4 (5.3)	9 (8.0)
Multiple	25 (17.2)	32 (20.0)	6 (8.8)	3 (7.7)	3 (2.7)	2 (2.6)	5 (4.4)

## Discussion

In this study, we investigated the detection of various respiratory pathogens over a two-year period following the lifting of restrictions and the declaration that COVID-19 is no longer a global public health emergency. The study offers several important insights from an epidemiological standpoint. The diversity of respiratory pathogens detected is notable. Rhinovirus/Enterovirus, RSV A&B, and Influenza A viruses were the most commonly identified pathogens, which aligns with global respiratory infection trends. However, the continued presence of SARS-CoV-2 underscores the evolving nature of respiratory infections and the need for ongoing surveillance and indicates that this virus will reach an adaptation within the human beings [26].

The monthly breakdown reveals distinct patterns in pathogen occurrences. This aligns with established knowledge in epidemiology that respiratory viruses often display seasonal peaks, typically during colder months [8, 27]. Understanding these temporal trends is crucial for preparedness and targeted interventions during high-transmission periods.

In this study, we used a multiplex PCR to assess multiple respiratory pathogens over a two-year period. The simultaneous evaluation of various pathogens and their temporal dynamics provides a holistic view of respiratory illness epidemiology within the studied population. Additionally, the detection of less common viruses (e.g., bocavirus, coronaviruses) showcases the breadth of the pathogen spectrum and highlights their low occurrence yet relevant presence. The study showed different respiratory pathogens detected among 1790 samples over two years. The prevalence of Rhinovirus/Enterovirus, RSV A&B, and Influenza A stands out as the most prominent pathogens. Influenza A/B constituted 172 (9.6%) and parainfluenza constituted (58, 3.2%). The Kingdom of Saudi Arabia established the Integrated Influenza Sentinel Surveillance System (IISS), its most sophisticated surveillance system to date, in 2022, in response to the COVID-19 pandemic and the effective execution of the National Influenza Surveillance Development Plan. There are 100 sites in the IISS (30 hospitals and 70 primary care clinics). It combines quick COVID-19 testing, training for healthcare providers, and on-site molecular testing for influenza and respiratory syncytial virus. For subtyping, genetic sequencing, and sharing, all test results are forwarded to the Public Health Laboratory at the Public Health Authority [28]. Various studies indicated that the detection of influenza among patients with ILI ranged from 3 to 12% in Saudi Arabia [29].

Bocavirus infection predominantly affected toddlers, while Adenovirus infection was more common in early childhood. In a previous study from Saudi Arabia, Bocavirus was seen in 1.6% of patients and most of them were diagnosed in the winter [30] and another study showed a prevalence of 11.2% in pediatric patients [31]. However, this virus was not detected among pilgrims [32]. The most common detected pathogen in this study was Rhinovirus/Enterovirus. Few studies in Saudi Arabia showed that Rhinovirus/Enterovirus affects children < 5 years of age and those less than two years [33, 34]. Of particular interest is the occurrence of RSV and Enterovirus/Rhinovirus in 2022–2023. The COVID-19 pandemic had a significant impact on RSV cases, leading to a decrease in the number of RSV cases and hospital admissions [16]. This decrease can be attributed to the implementation of infection control measures, similar to the decline observed in influenza cases in certain regions [16]. The impact on RSV activity during the pandemic has exhibited geographical variability, with certain countries reporting low transmission levels.  The impact of the pandemic on RSV activity has varied across different geographical areas. While some countries experienced low transmission during the pandemic, there has been an increase in RSV cases in subsequent years.  The absence of seasonal exposure to RSV during the pandemic may lead to a decline in population immunity, potentially increasing the risk of severe RSV infection. In a study from Egypt, a total of 530 patients under the age of 16 with ILI symptoms were enrolled and 49.1% of them tested positive for one or more of the three tested viruses, including 25.3% for influenza, 20.9% for RSV, and 2.6% for RSV-influenza coinfection [35].

The results of this study shed light on the prevalence of respiratory viruses in various age groups. The fact that rhinovirus is consistently ranked among the most detected pathogens in a variety of age groups indicates the important role that this pathogen plays. Rhinoviruses can cause a sore throat, cough, and nasal congestion, among other respiratory symptoms, and as a major cause of the common cold [36]. Previous studies support the high prevalence of RSV in infants and toddlers. RSV can cause severe respiratory illness, especially in infants, and is a leading cause of lower respiratory tract infections in young children. The results highlight the necessity of focused preventive initiatives, like immunization and better hygiene practices, within this vulnerable age group to reduce the burden of RSV infections [37]. The presence of influenza viruses across multiple age groups underscores the ongoing significance of influenza as a respiratory pathogen. Influenza infections can cause seasonal outbreaks and have the potential for severe complications, particularly among older adults and individuals with underlying health conditions. Vaccination remains a key strategy for reducing the impact of influenza, and efforts should be made to enhance vaccination coverage, especially in high-risk populations [38, 39]. The detection of SARS-CoV-2 in various age groups reflects the ongoing global pandemic and its impact on respiratory health. The higher prevalence of SARS-CoV-2 in young adulthood and middle adulthood highlights the susceptibility of these age groups to infection. It emphasizes the importance of continued adherence to preventive measures, such as vaccination, mask-wearing, and physical distancing, to mitigate the spread of COVID-19 and its potential long-term consequences [40].

The monthly occurrences of respiratory pathogens over the study period mirrors well-established seasonal patterns observed in respiratory virus activity. For instance, peaks in Rhinovirus/Enterovirus and RSV A&B detections in the colder months, particularly during late fall and winter. Similar findings were found in earlier Saudi Arabian research, which showed that most RSV infections happened in the winter [31, 41, 42]. These results were corroborated by a systematic review that concentrated on the Middle East and North Africa (MENA) region and showed that RSV was more common in the winter, specifically from November to February [21]. The observed variations in pathogen detections month-to-month underscore the complex interplay of factors such as climate, population immunity, behavior, and viral transmissibility.

The findings of this study hold implications for public health interventions. Understanding seasonal trends helps in optimizing vaccination strategies, preparing healthcare systems for potential surges, and implementing targeted preventive measures during high-risk periods. Moreover, the emergence of novel viruses like SARS-CoV-2 underscores the necessity for adaptable surveillance systems and rapid response strategies. In addition, study offers a valuable resource for further epidemiological studies. Longitudinal data on multiple respiratory pathogens can facilitate predictive modeling, epidemiological forecasting, and in-depth analyses of transmission dynamics, contributing to the advancement of public health strategies.

However, it is important to note few limitations of the study. Results might not be generally applicable because they are particular to the single center’s population and circumstances. The small sample size may impact the study’s statistical power and capacity to identify uncommon infections. Since the study was conducted over a two-year duration, seasonal variations in respiratory pathogen may not be fully captured. Certain cases or demographics that are more likely to seek treatment or surveillance at that specific center may be the focus of bias in the study. It is possible that some pathogens’ evolution, emergence, or shifts in prevalence during the study period were not fully recorded. External factors, like public health initiatives or community interventions, could have an impact on the study’s findings by influencing the spread of different pathogens.

In conclusion, the study showed continued emergence of RSV and Enterovirus/Rhinovirus as the predominant pathogens in 2022 and 2023. However, several other pathogens were detected at relatively low frequencies, including Influenza A, *Mycoplasma pneumoniae*, and *Legionella pneumophila.* The study emphasizes the importance of continuous surveillance, especially during seasonal shifts, to monitor the prevalence and circulation patterns of respiratory pathogens. The insights gleaned from such data can inform targeted public health strategies, including vaccination campaigns, infection control measures, and resource allocation, aimed at mitigating the burden of respiratory infections. Further studies are needed to be prospective and multi-centers and to have better studies concerning the detection and characterization of respiratory viruses, particularly those with the potential for emergence. Such studies would also require setting up a strategy for genotyping and/or sequencing of viruses with potential for emergence such as influenza and SARS-CoV-2.

## Data Availability

Available upon a reasonable request.
